# Kinetic Model of Nav1.5 Channel Provides a Subtle Insight into Slow Inactivation Associated Excitability in Cardiac Cells

**DOI:** 10.1371/journal.pone.0064286

**Published:** 2013-05-16

**Authors:** Zheng Zhang, Zhiwen Zhao, Yongfeng Liu, Wei Wang, Ying Wu, Jiuping Ding

**Affiliations:** Key Laboratory of Molecular Biophysics of the Ministry of Education, School of Life Science and Technology, Huazhong University of Science and Technology, Wuhan, Hubei, China; Virginia Commonwealth University, United States of America

## Abstract

Voltage-gated sodium channel Nav1.5 has been linked to the cardiac cell excitability and a variety of arrhythmic syndromes including long QT, Brugada, and conduction abnormalities. Nav1.5 exhibits a slow inactivation, corresponding to a duration-dependent bi-exponential recovery, which is often associated with various arrhythmia syndromes. However, the gating mechanism of Nav1.5 and the physiological role of slow inactivation in cardiac cells remain elusive. Here a 12-state two-step inactivation Markov model was successfully developed to depict the gating kinetics of Nav1.5. This model can simulate the Nav1.5 channel in not only steady state processes, but also various transient processes. Compared with the simpler 8-state model, this 12-state model is well-behaved in simulating and explaining the processes of slow inactivation and slow recovery. This model provides a good framework for further studying the gating mechanism and physiological role of sodium channel in excitable cells.

## Introduction

Nav1.5, the cardiac isoform of the voltage-dependent sodium channel α subunit, is encoded by the human SCN5A gene which locates on chromosome 3p21 [1]. In the firing process of cardiac action potentials (APs), Nav1.5 channel carries a rapid inward Na^+^ current (I_Na_) in response to a depolarization and then goes into “a fast inactivated state” with a small fraction of remnant currents throughout the whole process of depolarization. This persistent current termed as a late I_Na_ plays a critical role in the heart [2]–[4]. After that, inactivated channels recover from the process of repolarization and prepare for the next process of depolarization. The generalized morphology of myocardial APs in humans and other large mammalian species contains a rapid upstroke followed by a depolarized plateau potential lasting for more than 100 ms [5], [6], whereas the AP in neurons usually consists of a rapid upstroke and an immediate repolarization [7]. With the prolonged depolarization, Nav1.5 channels progressively enter “a slow inactivated state”, corresponding to a slow recovery process with the time constants ranging from hundreds of milliseconds to several seconds [8], [9]. Slow inactivation substantially suppresses Na^+^ currents to control the cell excitability. Mutants which cause enhanced slow inactivation are often associated with several clinical heart diseases [10]–[13]. However, the gating mechanism of slow inactivation of Nav1.5 remains elusive.

Over the past 15 years, numerous mutations in SCN5A have been reported to be associated with various rare arrhythmia syndromes, such as congenital Long QT syndrome type 3 (LQTS3), Brugada syndrome (BrS), progressive cardiac conduction defect (PCCD), sick sinus syndrome (SSS) and arterial standstill [10]–[14]. To better understand the linkage between the gating of the Nav1.5 channels and heart diseases, we think that one feasible way is to use kinetic models. The dominant paradigm for modeling voltage-gated ion channel kinetics over the past 60 years has been dependent on the giant squid axons experiments of Hodgkin and Huxley [15]. Since then, the H-H models have been extensively used in data analysis of cellular electrophysiology. However, with the availability of high resolution data, many ion channels exhibit features beyond the traditional H-H models [16], [17]. As a consequence, more complicated Markov models have been proposed for analyzing the ion-channel kinetics [18]–[21]. Such models produce more precise description to the ion-channel kinetics, which can be ultimately used to understand the firing properties of APs in excitable cells.

In this study, we proposed a two-step inactivation Markov model for simulating the Nav1.5 currents including the slow inactivation and bi-exponential recovery.

This work provided a solid basis for studying the detailed gating mechanism and the electrophysiological role of sodium channel in excitable cells.

## Materials and Methods

### Cell culture and transfection

The full-length cDNAs for human Nav1.5 (SCN5A) was subcloned into pcDNA3.1 Zeo(+) (Clontech). The construct was verified by DNA sequencing. HEK293 cells were cultured in Dulbecco's modified Eagle's medium (DMEM) supplemented with 10% fetal bovine serum (FBS) and incubated at 37°C in 5% CO_2_. One day before transfection, cells were transferred to 24-well plates. At 90% confluence, cells were transiently transfected using Lipofectamine2000 (Invitrogen). Electrophysiological experiments were performed at 1–2 days after transfection.

### Electrophysiology

The whole-cell mode was only used in all experiments. Patch pipettes were pulled from borosilicate glass capillaries with a resistance of 1.5–2.5 MΩ, after filled with pipette solution. The series resistances were compensated with 80%–90%. All the experiments were performed with a patch clamp amplifier (Axopatch 200B, Axon Instruments, Union City, Calif., USA) with its software (Clampex) at room temperature (23–25°C). The currents were typically digitized at 10–100 KHz and filtered at 5 kHz (Fig. S1). The pipettes solution contained (in mM): 140 CsCl, 10 NaCl, 10 HEPES, 1 EGTA, pH 7.4, adjusted with CsOH. The bath solution contained (in mM): 140 NaCl, 5 KCl, 2 CaCl_2_, 1 MgCl_2_, 10 HEPES, pH 7.4, adjusted with NaOH.

### Data Analysis

Patch clamp recording data were analyzed with Clampfit (Axon Instruments, Inc.) and Sigmaplot (SPSS, Inc.) software. Unless otherwise stated, the data are presented as mean ± S.D..

The conductance-voltage (G–V) curves for activation were fitted to the a Boltzmann equation as below, 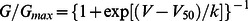
(1) where *V*_50_ is the half maximal voltage, *G* the conductance, *G_max_* the maximum conductance and *k* the slope factor.

The steady-state inactivation was fitted to a Boltzmann equation as below, 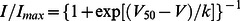
(2) where *V*_50_ is a half availability voltage, and *k* is the slope factor.

Development of slow inactivation was fitted to a single exponential function as below, 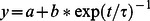
(3) where *τ* is time constant, *a* and *b* are the partition coefficients.

Recovery curves were fitted to the mono-exponentail or bi-exponential equations as below, 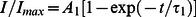
(4)

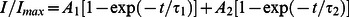
(5) where *I* is the peak current, *I_max_* the maximal peak current, *A_1_* and *A_2_* the proportional coefficients, *t* the time, *τ_1_* and *τ*_2_ the fast and slow recovery time constants, respectively.

### Mathematical modeling and simulation

The differential equations for the kinetic modeling were solved numerically, using a QMatrix or Five-order Runge-Kutta integration method. The fitting procedure is bad on a PSO-GSS algorithm for direct estimation of rate constants from macroscopic currents [22]. The integrating routines were written and executed with software CeL (HUST, Wuhan, Hubei, China), compiled with the C++ compiler to run under Windows XP [23]. Kinetic parameters were optimized with CeL as previously described. [22]

## Results

### Models for Nav1.5 channels

To understand the gating mechanism of Nav1.5, the currents of activation, steady-state inactivation and deactivation are absolutely necessary to provide us the detailed information of rate constants required for a kinetic model. In this study, we are to provide a detailed description on the behavior of Nav1.5 currents arising from expression of *SCN5A* α-subunits in HEK293 cells, to present a kinetic model that appears to account for the observed currents of Nav1.5, and finally to construct a cardiac model cell with the built-in Nav1.5 channels to evaluate its physiological role in cardiac cells.

In models, the activation current depends on the forward (activation) rates from the closed (C) to the open (O) to the inactivated (I) states; the deactivation current depends on the backward (deactivation) rates from O to C; the steady-state inactivation current depends on the rates from O to I or C to I. Although currents rely on all the rates in model, each pathway is predominantly influenced by a certain combination of rates. Therefore, the model with all the rates can be finally determined from those currents [22].

### Kinetic properties of Nav1.5 channels

**Figure 1:**
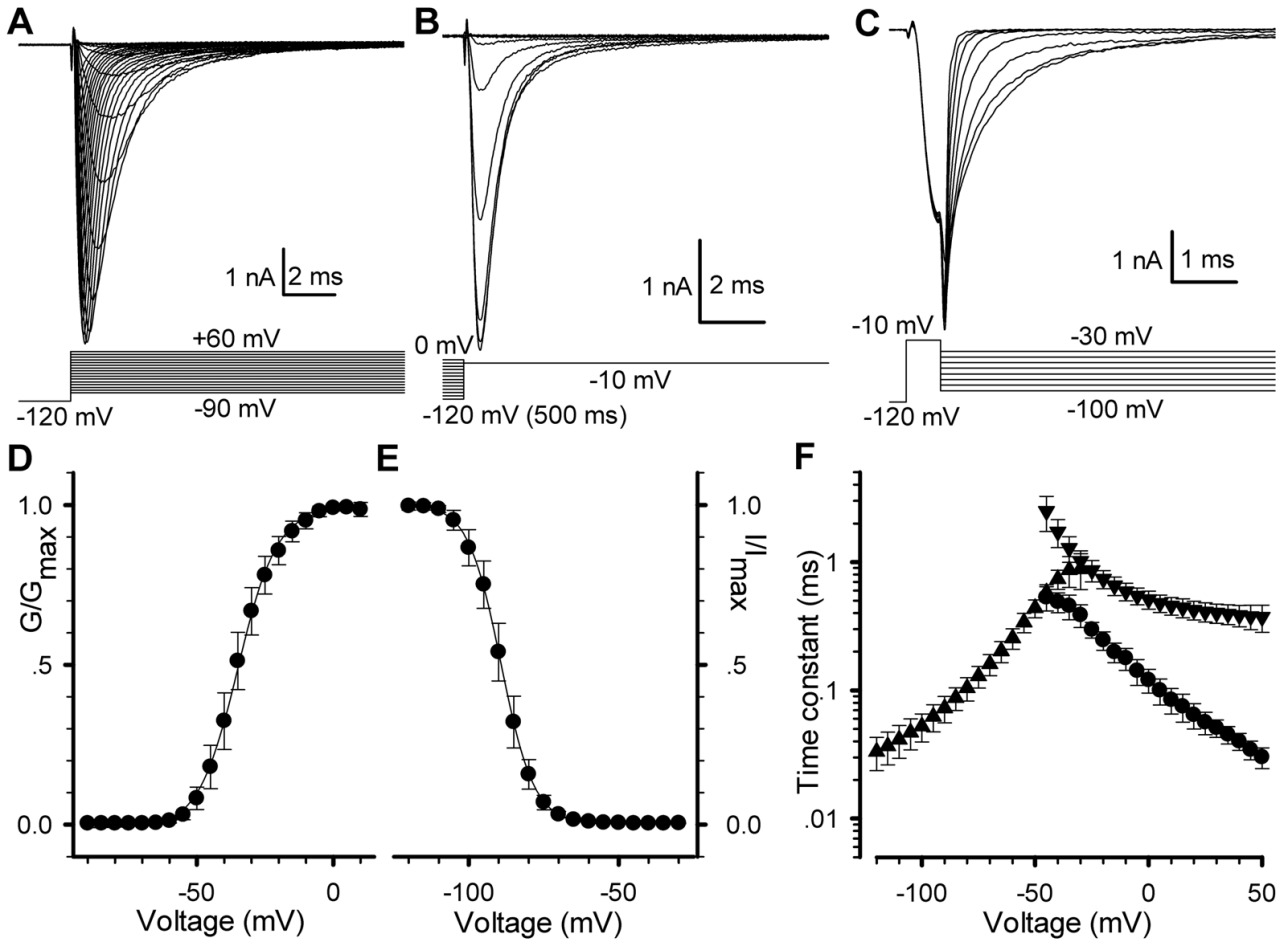
The currents and kinetic characteristics of voltage-gated sodium (Nav1.5) channels expressed in HEK293. (A–C) The representative currents of Nav1.5 channels were produced with the voltage protocols placed at the bottom, respectively. (D and E) The G–V curve (D) and channel availability curve (E) were measured from (A) and (B), respectively. Normalized curves were fitted to the Eq. 1 and Eq. 2. For activation, V_50_ = −34.5±1.5 mV (*P*<0.001) and *k* = 7.2±0.6 (n = 14); for steady-state inactivation (availability), V_50_ = −89.1±1.6 mV and *k* = 5.5±0.4 (n = 11). Error bars represent S.D.. (F) Time constants are plotted as a function of membrane potentials. The time constants of activation (circle) and inactivation (triangle down) and deactivation (triangle up) were derived from mono-exponential fits to the data shown in (A and C).

A set of the whole-cell experiments was thus performed on Nav1.5-transfected HEK293 cells for collecting the kinetics of activation (Fig. 1A), steady-state inactivation (Fig. 1B) and deactivation (Fig. 1C). In Fig. 1A, Nav1.5 currents exhibit a rapid activation and then a completed inactivation by a 20 ms depolarizing voltage steps ranging from−90 to+60 mV in 5 mV increments after a holding potential of−120 mV to remove the possible inactivation. The activation and inactivation processes of Nav1.5 in Fig. 1A can be described by C→O→I. Thus, the forward rates can be determined by fitting it to the activation currents. Deactivation currents were acquired by a 0.25 ms pulse to−10 mV following a holding potential to−120 mV and ending with a 20 ms voltage steps from−100 to−30 mV in 10 mV increments (Fig. 1C). Similarly, the backward rate of C←O can be determined by fitting it to deactivation currents. The voltage dependence of steady-state inactivation currents, standing for the availability of channels, was obtained by applying a set of conditioning voltages ranging from−120 to 0 mV for 500 ms in 10 mV increments and then measured at−10 mV with a 20 ms test pulse as indicated in Fig. 1B. The backward rates of C←O←I can be determined by fitting them to the corresponding currents.

The normalized G–V curve of Nav1.5 confers an averaged value of V_50_ = −34.5±1.5 mV (Fig. 1D). The V_50_ for the steady-state inactivation (availability) of 500 ms is−89.1±1.6 mV (Fig. 1E). These gating results are consistant with previous work for Nav1.5 [24]. Fig. 1F shows the time constants of activation (τ_a_), deactivation (τ_d_) and inactivation (τ_i_) of Nav1.5 channels.

**Figure 2:**
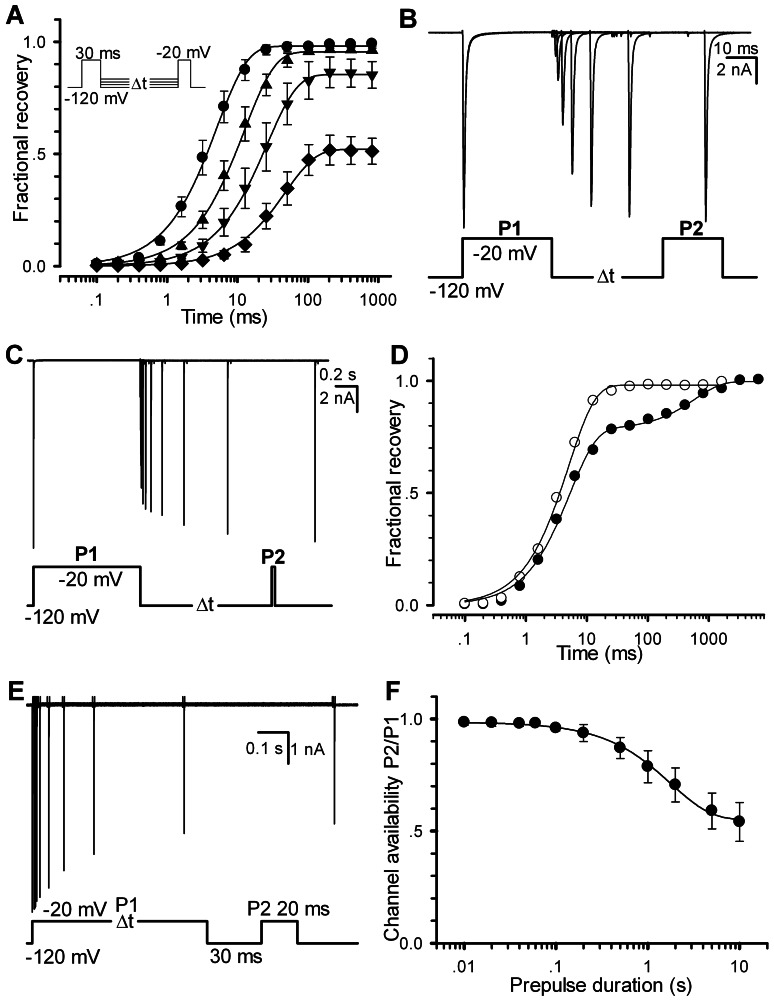
Recovery property of Nav1.5. (A) Fractional recovery from inactivation was performed with the voltage protocol in inset. Time constants of recovery are 5.1±0.9 (n = 9), 12.5±2.1 (n = 7), 26.1±3.8 (n = 6) and 47.9±3.4 (n = 6) ms at−120,−110,−100 and−90 mV, respectively. (B–C) Traces were obtained from the same patch at−120 mV with P1 = 30 ms for (B) and P1 = 1000 ms for (C). (D) The fractional recovery curves are plotted for (B) and (C), respectively. The solid lines are fits to the mono-exponential Eq.4 (empty circle) for (B) and the bi-exponential Eq. 5 (solid circle) for (C). (E) The slow inactivation currents were evoked by the voltage protocol with the various P1 durations shown at the bottom. (F) Development of slow inactivation. The solid line is a fit to Eq. 3 with a time constant τ = 1.79±0.11 s (n = 7). For all of cases, P2 = 20 ms.

Alternatively, the channel availability can be obtained by directly measuring the recoveries of Nav1.5. In this study, a two-pulse (prepulse P1 and test pulse P2) protocol was used for all of recovery experiments. Here we define the fractional currents as a ratio of I_i_(P2, V, t_i_)/I(P1, −120 mV). Here I is the current, P1 the prepulse duration, P2 the test pulse duration, V the recovery voltage and t_i_ the ith time interval between P1 and P2. Fig. 2A displays a set of fractional recovery curves arising from a two-pulse protocol (P1 = 30 ms, P2 = 20 ms, V = −120,−110,−100 and−90 mV). The recovery time constants are 5.1±0.9, 12.5±2.1, 26.1±3.8 and 47.9±3.4 ms for−120,−110,−100 and−90 mV, respectively. A typical experiment exhibited a mono-exponential recovery (Fig. 2B). Especially, when t_i_ = ∞, a set of the fractional ratio I_i_(P2, V, t_i_)/I(P1,−120 mV) will tend to their steady-state values, which can confer a steady-state inactivation curve. When P1 = 1000 ms, however, it shows a bi-exponential recovery (Fig. 2C), suggesting that a secondary (slow) inactivation state exists. Compared with the single-recovery curve (−120 mV), the double-recovery curve (−120 mV) is plotted in Fig. 2D. For P1 = 30 ms (empty circle), the recovery time constant is 5.0 ms; for P1 = 1000 ms (solid circle), the fast recovery time constant (τ_r-fast_) is 5.2 ms (78%) and the slow one (τ_r-slow_) is 596.3 ms (22%). To further explore the effect of P1 duration, a development of slow inactivation was executed. Cells were depolarized by a−20 mV prepulse P1 with various durations to elicit inactivation, followed by a−120 mV interpulse with only a 30 ms duration, presumably to remove the recovery from fast inactivation (Fig. 2E). The remaining peak currents of the test pulse P2 measured at−20 mV confers a time constant of 1.79±0.11 s (Fig. 2F), suggesting that there is a slow inactivation component in Nav1.5 channels.

### The kinetic model of Nav1.5 channels

To better understand the gating mechanism of Nav1.5, it is necessary to construct a Markov model for precisely matching all of the Na^+^ currents shown in this study. After that, we aim to further explore the physiological role of Nav1.5 in cardiac cells.

**Figure 3:**
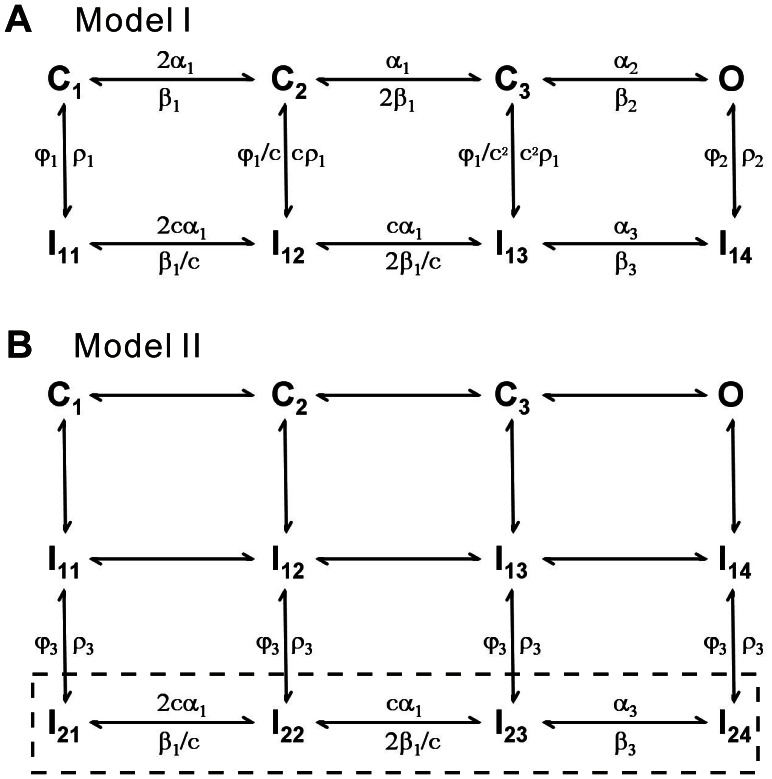
Kinetic models of Nav1.5. (A) The 8-state Model I has three closed states (C_1_, C_2_, and C_3_), four inactivated states (I_11_–I_14_) and one open state (O). (B) Model II has four additional slow inactivated states (I_21_–I_24_) attached to Model I. The values of parameters in both the models are given in Table 1.

At first, a typical 12-state model, composed of five closed states, six inactivated states and one open state, was used as the Nav1.5 model [25], However we failed to get a good fit to the present data. Alternatively, we considered the formalized Nav model of m^3^h proposed by Hodgkin and Huxley [15], where m is an activation factor and h an inactivation factor. Actually, m^3^h has an open probability equal to that of an 8-state Markov kinetic model [21]. Mimicking the Hodgkin-Huxley (H-H) model, an 8-state model, composed of three closed (C_1_–C_3_) states, four inactivated (I_11_–I_14_) states and one open (O) state, was chosen to simulate the Nav1.5 currents (Fig. 3A, Model I). Differing from the previous models, all rates are voltage-dependent in this model. The reason for this change was to make model more flexible and obtain the highest score evaluated by the software CeL [22]. For slow inactivation, we constructed a new model (Model II) by adding the four slow inactivated states I_21_–I_24_ to I_11_–I_14_ in Model I as shown in the boxed region (Fig. 3B). In this model, we assume that the occupancies of I_11_–I_14_ transits into I_21_–I_24_ so slowly as to lead a slow inactivation. The parameter values of two models are listed in Table 1.

**Table 1:** Best fit model parameters.

	k	n
α_1_	9.435	39.70
α_2_	441.1	6.593
α_3_	11.17	11.64
β_1_	0.000037	−7.770
β_2_	0.2241	−21.13
φ_1_	0.000020	−13.07
φ_2_	0.000302	−47.08
φ_3_	0.000230	−57.21
ρ_2_	1.823	92.78
ρ_3_	0.000315	965.2
g	0.01296	
a	85.62	
f	15.64	
c	2.146	

Each of rate constants has the expression x = k * exp^V/n^ except the ρ_1_ = g/(1+exp(−(v+a)/f)) and the cyclic balancing rates β_3_, calculated from microscopic reversibility of cycles. Here the rate x and the pre-exponential factors k and g are in ms^−1^ and the exponential factor n, a, f and voltage V (trans-membrane voltage) in mV^−1^. The parameter c is non-dimensional.

**Figure 4:**
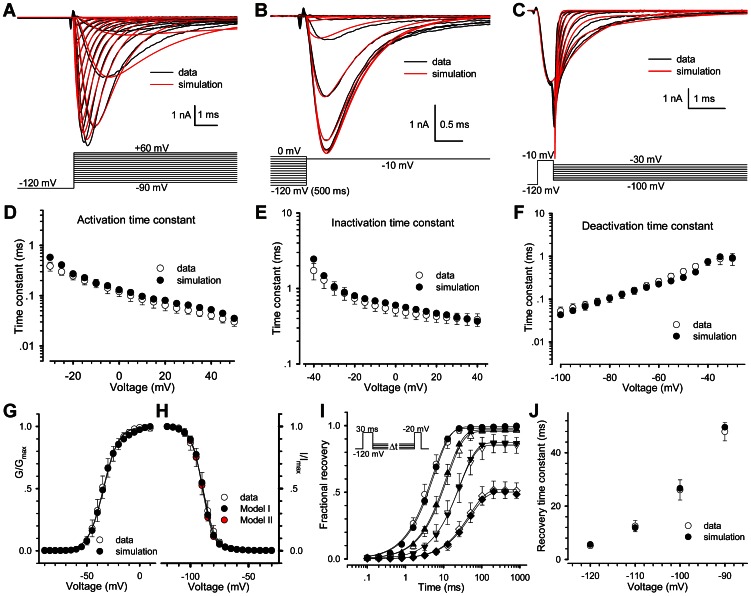
Comparison of kinetic characteristics between data and simulations. Unless otherwise stated, simulation was derived from Model I. (A–C) Fitting the Model I to the data of activation (A), steady-state inactivation (B) and deactivation (C). Black traces represent data and red ones simulations. (D–F) Time constants for activation (D), fast inactivation (E) and deactivation (F) are plotted as a function of voltages. The empty and solid circles represent the data and simulations as indicated. (G) The V_50_′s values of G–V curves of activation are 34.5 mV for data and−34.0 mV for simulation. (H) The V_50_′s values of steady-state inactivation are−89.1 mV for data,−89.2 mV for Model I and−89.7 mV for Model II. (I) Fractional recoveries of Nav1.5 were acquired at−120,−110,−100 and−90 mV with P1 = 30 ms and P2 = 20 ms. (J) Time constants of recovery of data and simulation are 5.1 and 5.6, 12.5 and 11.9, 26.1 and 26.6 and 47.9 and 49.6 ms at−120,−110,−100 and−90 mV, respectively.

In Fig. 4A–C, the Model I replicates the activation, deactivation and steady-state inactivation of Nav1.5, indicating that it is capable to be a Nav1.5 model. The time constants of data and simulation derived from Fig 4A–C are near overlapped (Fig. 4D–F). The V_50_ of activation is−34.5 mV for data and−34.0 mV for simulation (Fig. 4G); the V_50_ of steady-state inactivation is−89.1 mV and−89.2 mV (Fig. 4H). Recovery for the short P1 of 30 ms could also be replicated by Model I (Fig. 4I, J). Time constants from−120 to−90 mV are 5.1 and 5.6, 12.5 and 11.9, 26.1 and 26.6, 47.9 and 49.6 ms for data and simulation, respectively.

However, Model I failed to account for the slow recovery process. We realized that this insufficiency was related to the structural constraint of Model I. According to this 8-states model, it can not account for the bi-exponential recovery process, which has the time constants τ_r-fast_ = 5.2 ms and τ_r-slow_ = 596.3 ms with a nearly 100 times difference (Fig. 2). Interestingly, the time constant (τ_r-fast_) of fast component remains no change, compared with that of single recovery, suggesting that it is better to insert a secondary (slow) inactivation pathway to model I. The simplest way is obviously to directly add one more inactivated state I_s_ (or I_slow_) to open state (O) [7], [26]. Essentially, this I_s_ is to share a certain amount of probability with the original I_f_ (or I_fast_) state. It will slowly go and back between I_s_ and O states as a slow component of recovery. Unfortunately, this modified model was incapable to account for the duration-dependent inactivation or bi-exponential recovery in our trials.

**Figure 5:**
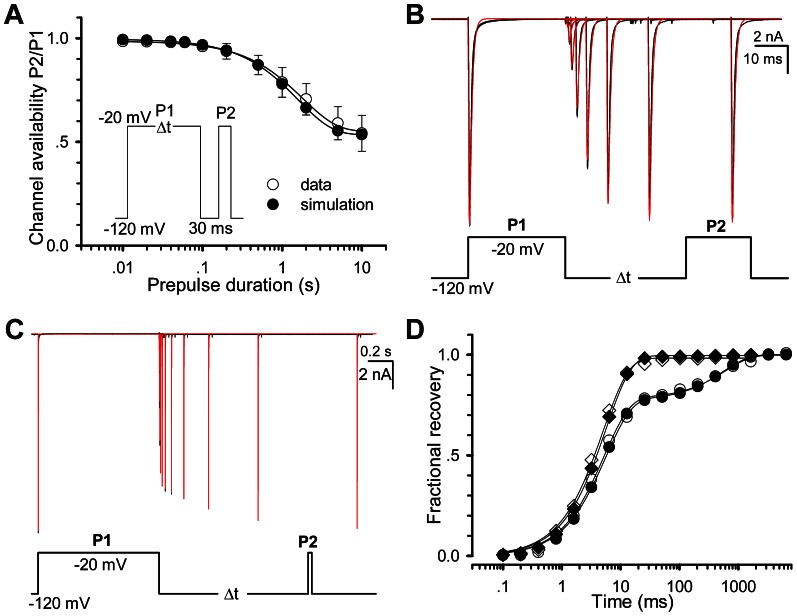
Comparison of Slow inactivation and recovery between the data and simulations. The empty and solid circles represent respectively the data and simulations. (A) Development of slow inactivation. The solid lines are fits to Eq. 3 with the time constants of 1.79 s (data) and 1.58 s (simulation). (B–C) Fitting the Model II to the data shown in Fig 2B–C. Black traces are data and red ones fits. (D) The curves of fractional recovery are plotted for data and simulations in (B) and (C). Square and circle are for P1 = 30 ms in (B) and P1 = 1000 ms in (C).

After trying several different models, we finally determined to use the 12-state two-step inactivation model named as Model II (Fig. 3B). Based on the structure of Model II, the slow inactivation of channels should undergo two steps during the depolarization process, for simplicity, first from O to I_14_ (fast) and then to I_24_ (slow). Upon repolarization, the fractional occupancy residing in the fast inactivation state I_14_ transfers quickly from I_14_ to C_1_ mainly via I_13_, I_12_ and I_11_, while the fraction residing in the slow inactivation state I_24_ transfers slowly from I_24_ to C_1_ mainly via I_24_, I_23_, I_22_, I_21_ and I_11_. Prolonging the duration of depolarization, more channels go to I_24_ from I_14_ and more channels have to recovery via a slow pathway. The rates between I_1x_ and I_2x_ are so small that Model II fitted nearly the same results to Model I in short-duration processes (Fig 4A, C–G, I–J). There is only a slight difference on fits, comparing the Model II with Model I (Fig 4B and H). However, Model II was also well-behaved in simulating the long P1 duration case. More kinetics of Model II is shown in Fig 5. The development curve of slow inactivation is shown in Fig 5A. Time constants are 1.79 s for data and 1.58 s for simulation. Moreover, all the fits (red) generated by the Model II in Fig. 5B–C are coincident with the recovery traces (black) shown previously in Fig 2B–C, suggesting that the Model II can also well replicate the whole processes including the slow recovery. For the short P1 of 30 ms, recovery time constants are 5.0 ms and 5.6 ms for data and simulation, respectively; for the long P1 of 1000 ms, are 5.2 ms (fast, 78%) and 596.3 ms (slow, 22%) for data, 5.6 ms (fast, 77%) and 557.2 ms (slow, 23%) for simulation (Fig. 5D).

## Discussion

In this study, a novel 12-state two-step inactivation kinetic model was successfully developed to study the slow inactivation mechanism of Nav1.5 channels, especially to illustrate the mechanism of duration-dependent bi-exponential recovery based on a set of Nav1.5 currents recorded by patch-clamp experiments.

The proposed model II (or two-step inactivation model) can be regarded as a hierarchical structure. Firstly, Model I well duplicated the activation, deactivation, steady-state (fast) inactivation and fast recovery (Fig. 4). Three closed states in Model I correspond to the *m* ^3^ Hodgkin-Huxley formalism, previously suggested by Markov models [18], and FRET experiments [27], [28]. Especially, the latter suggested that only three major protein conformations were required for ion permeation to occur. Secondly, Model II well described all the Nav1.5 kinetics including the slow inactivation and slow recovery, the mission impossible for Model I (Fig. 5).

The slow inactivation process in Model II can be briefly summarized as O←→I_1_←→I_2_, named as two-step inactivation model. Here I_1_ and I_2_ are the fast and slow inactivation states, respectively. In contrast, Jarecki et al. used an alternative two-state inactivation model I_2_←→O←→I_1_ to investigate the resurgent sodium currents [29]. We tested this model and found that it did not work well in explaining both the inactivation and the slow recovery at variety of voltages. Therefore, the two-step inactivation model should be more appropriate to our work.

HEK293 cell has endogenous voltage-gated Na channels such as Nav1.7 and voltage-gated Ca2+ channels [30]. But the endogenous currents of HKE293 in different labs were variable because of different generation, environment, or other factors. We used cesium instead of potassium in our pipette solution (see in Method) in order to block the endogenous potassium current of HEK293. Secondly, we used 100 nM TTX as blockers (Fig. S2A). The change of Nav1.5 current was very small, which means there was no significant Nav1.7 current (TTX sensitive) in our HEK293 cells. Then we tested the currents of un-transfected cells. A large proportion of results revealed that there was no significant endogenous currents. Other proportion of results showed endogenous inward current with amplitude of 20 to 40 pA (Fig. S2B), which was less than one percent of transfected Nav1.5 current described in our work.

It is important for this discussion to point out that sodium channel (including α and β subunit) functions are critically dependent on the particular heterologous expression system used [31]. Current data sometimes are conflicting, possibly due to differences in experimental conditions or, more importantly, species studied [32]. In Xenopus oocytes, for instance, injection of the α subunit cRNA shows an abnormally large component of a intermediate inactivation mode [33]–[35], which is substantially reduced by co-injection of cRNA encoding the β1 subunit [34]. In contrast, the alone expressed α subunit in HEK293 has shown a kinetic behaviour similar to that of the native preparations [36], [37]. This is due to an abundant endogenous expression of mRNA encoding the β1A subunit in HEK293 [38]. The endogenous β1A subunit is sufficient for suppressing the intermediate inactivation of sodium currents by co-assembly with α-subunits [38]. Though the kinetics of our Nav1.5 model may have slight difference compared with other results obtained from variant tissues or species,

the mechanism of slow inactivation should be the similar. Our model can be also applicable to the data from other tissues or species with proper adjustment in parameters. As mentioned previously, some mutations associated with heart diseases, e.g. BrS or PCCD, often occur from the enhanced slow inactivation of Nav1.5 [10], [39]–[42], as it suppresses the Na^+^ current (loss of function) to decrease the excitability of cardiac cells. In other words, a loss of Na^+^ current prevents the membrane potential from reaching the threshold of AP, thereby slowing conduction. Therefore, our work provides a useful tool to investigate the linkage between the slow inactivation of Nav1.5 and clinical heart diseases.

Considering that there is a high similarity in the Nav1.x family, we believe that the two-step inactivation model is applicable to the other sodium channels with their mutations, after making proper changes in parameters. All of quantitative analyses were made possible by recent advances in kinetic modeling algorithms and software (CeL) that allowed us to quickly construct and fit models with large amounts of states from the comprehensive data ranged from 5 ms to more than 10 s [22]. Therefore, this work provides a convenient platform for further investigating the detailed linkage between the sodium channels and diseases in the future.

## Supporting Information

Figure S1 **The kinetic comparison of Nav1.5 channel with diverse frequency of filtration.** (A–C) Time constants for activation (A), fast inactivation (B) and deactivation (C) are plotted as a function of voltages. The diverse colors of circles represent the diverse frequency of filtration, red 2 KHz, green 10 KHz, blue 100 KHz and black 5 KHz, as indicated. Current traces are shown in (D). Red traces represent 10 KHz and black ones represent 5 KHz.
(TIF)

Figure S2 **The endogenous currents of HEK293 cell.** (A) Nav1.5 current before and after addition of 100 nM TTX. (B) Endogenous inward current of un-transfected HEK293 cell.
(TIF)
