# Supplementary figures and images for: Kinetic Model of Nav1.5 Channel Provides a Subtle Insight into Slow Inactivation Associated Excitability in Cardiac Cells
Source: PLoS One. 2013 May 16;8(5):e64286. doi: 10.1371/journal.pone.0064286 (PMC3655986; doi:10.1371/journal.pone.0064286)

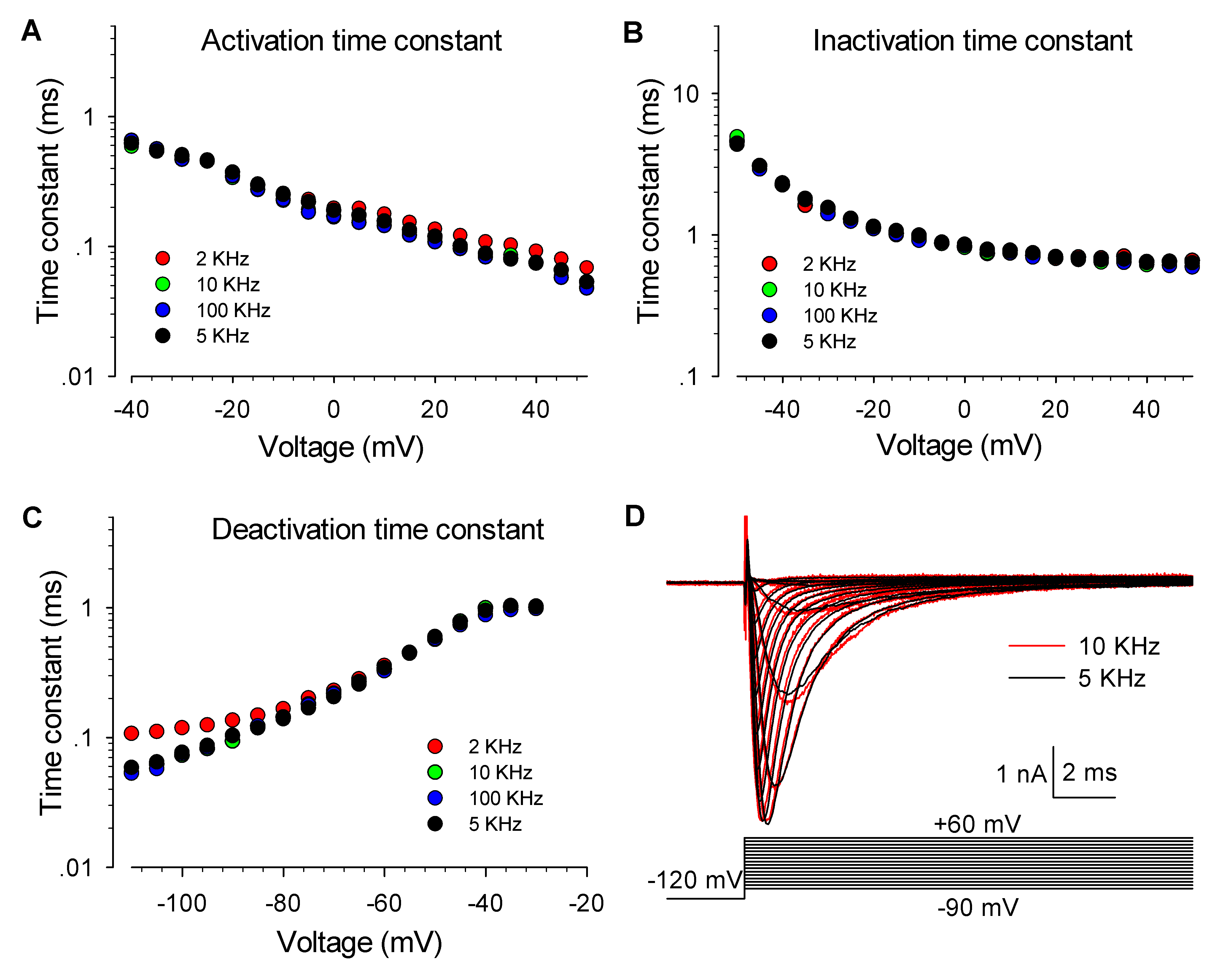

Supplement: Figure S1 — The kinetic comparison of Nav1.5 channel with diverse frequency of filtration. (A–C) Time constants for activation (A), fast inactivation (B) and deactivation (C) are plotted as a function of voltages. The diverse colors of circles represent the diverse frequency of filtration, red 2 KHz, green 10 KHz, blue 100 KHz and black 5 KHz, as indicated. Current traces are shown in (D). Red traces represent 10 KHz and black ones represent 5 KHz. (TIF) [file pone.0064286.s001.tif]

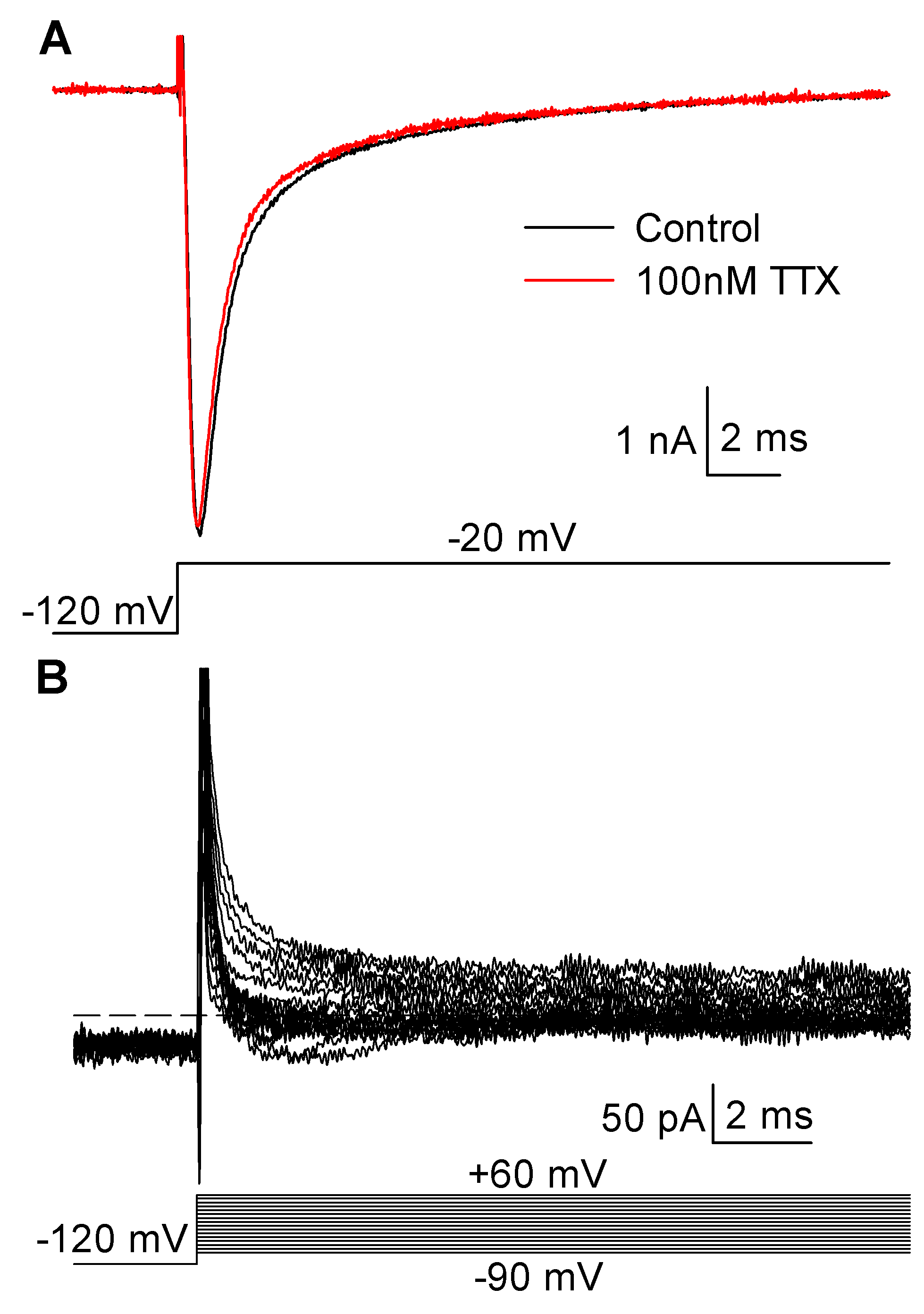

Supplement: Figure S2 — The endogenous currents of HEK293 cell. (A) Nav1.5 current before and after addition of 100 nM TTX. (B) Endogenous inward current of un-transfected HEK293 cell. (TIF) [file pone.0064286.s002.tif]
